# Asymmetric Response of Costa Rican White-Breasted Wood-Wrens (*Henicorhina leucosticta*) to Vocalizations from Allopatric Populations

**DOI:** 10.1371/journal.pone.0144949

**Published:** 2015-12-15

**Authors:** Teresa M. Pegan, Reid B. Rumelt, Sarah A. Dzielski, Mary Margaret Ferraro, Lauren E. Flesher, Nathaniel Young, Alexandra Class Freeman, Benjamin G. Freeman

**Affiliations:** 1 Department of Ecology and Evolutionary Biology, Cornell University, Ithaca, NY, United States of America; 2 Cornell Laboratory of Ornithology, Ithaca, NY, United States of America; National Cheng-Kung University, TAIWAN

## Abstract

Divergence in song between allopatric populations can contribute to premating reproductive isolation in territorial birds. Song divergence is typically measured by quantifying divergence in vocal traits using audio recordings, but field playback experiments provide a more direct way to behaviorally measure song divergence between allopatric populations. The White-breasted Wood-Wren (*Henicorhina leucosticta*; hereafter “WBWW”) is an abundant Neotropical species with four mitochondrial clades (in Central America, the Darién, the Chocó and the Amazon) that are deeply divergent (~5–16% sequence divergence). We assessed the possibility that the WBWW as currently defined may represent multiple biological species by conducting both statistical analysis of vocal characters and field playback experiments within three clades (Central America, Chocó and Amazon). Our analysis of vocal traits revealed that Central American songs overlapped in acoustic space with Chocó songs, indicating vocal similarity between these two populations, but that Central American songs were largely divergent from Amazonian songs. Playback experiments in the Caribbean lowlands of Costa Rica revealed that Central American WBWWs typically responded aggressively to songs from the Chocó population but did not respond to playback of songs from the Amazonian population, echoing the results of the vocal trait analysis. This marked difference in behavioral response demonstrates that the songs of Central American and Amazonian WBWWs (but not Central American and Chocó WBWWs) have diverged sufficiently that Central American WBWWs no longer recognize song from Amazonian WBWWs as a signal to elicit territorial defense. This suggests that significant premating reproductive isolation has evolved between these two populations, at least from the perspective of the Central American population, and is consistent with the possibility that Central American and Amazonian populations represent distinct biological species. We conclude by advocating for the further use of field playback experiments to assess premating reproductive isolation (and species limits) between allopatric songbird populations, a situation where behavioral systematics can answer questions that phylogenetic systematics cannot.

## Introduction

Ecologists, evolutionary biologists and conservation biologists all require rigorous taxonomies to conserve species and understand ecological and evolutionary patterns and processes. Thus, a central challenge in biodiversity science is to determine species limits. This is often addressed using “species concepts”, which are simple ways of viewing the complex processes that result in speciation. The Biological Species Concept (BSC) proposes that species are defined based on gene flow, and has historically been the prevailing species concept in use for birds and other sexually reproducing taxa [1,2]. The BSC assumes that species have evolved some level of premating and/or postmating reproductive isolation, and is therefore easiest to apply when taxa have overlapping distributions, as morphological traits, genetic data and behavioral observations can typically determine whether such taxa reproduce with each other or not. However, speciation in birds typically occurs in allopatry [2,3]. Because secondary contact can take millions of years to occur [4,5], most groups of closely related taxa therefore have allopatric distributions. In these cases, populations do not physically come into contact. Intrinsic reproductive isolation—isolation caused by something other than physical separation—may occur in allopatry when selection and genetic drift act on traits that are important for successful interbreeding [1]. To test whether allopatric species have evolved reproductive isolation, researchers must use measurements of genotypic and phenotypic divergence to infer whether gene flow would occur if their populations were in contact [6]. Genetic divergence can be used to determine species limits if the evolution of reproductive isolation is correlated with evolutionary time such that species with large genetic distances (e.g., > 4% sequence divergence in mitcochondrial DNA (mtDNA) [7]) are unlikely to experience gene flow if they come into secondary contact. However, this may not always be the case [8] and there is some evidence that the evolution of reproductive isolation is particularly slow in the tropics [9].

Ornithologists analyzing species limits in closely related allopatric taxa commonly attempt to assess divergence in traits associated with premating barriers to reproductive isolation between allopatric populations [10–12]. In particular, vocal traits (e.g., songs) and plumage traits are important cues that influence mating decisions in birds, and divergence in these characters between allopatric populations provides evidence that they would not interbreed should they come into secondary contact [6]. Analyses of vocal traits are widely used to inform species limits, both in suboscine passerines, which generally do not learn songs [13], and also in the more speciose clade of oscine passerines. The inferential ability of these analyses may be limited in oscines, which can learn songs: therefore song differences can be culturally transmitted as well as genetically transmitted in this clade [14,15]. These analyses assume that the vocal cues birds use when making mating decisions are the same characteristics that researchers measure from audio recordings. While this assumption may generally be valid, it is also possible in many cases to directly observe this behavior in the field—researchers can simulate the presence of an allopatric individual by presenting wild birds with audio playback of songs from allopatric populations. Such playback experiments are a powerful but underused method to infer species limits between allopatric populations of birds, and allow researchers to directly assess the behavioral responses of birds to a simulated instance of secondary contact [16–18].

**Fig 1:**
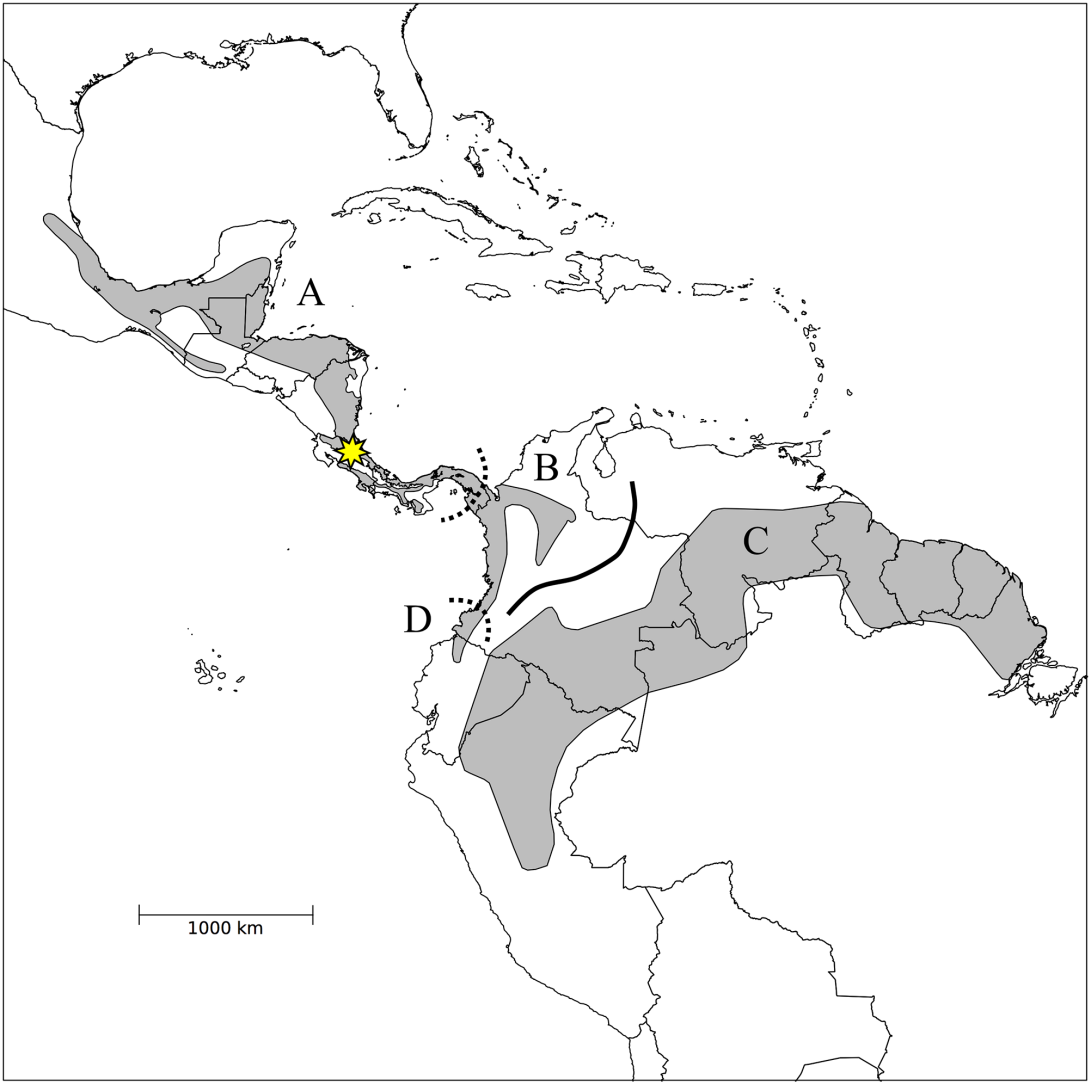
Range map of the White-breasted Wood-Wren. This map illustrates the distributions of four deeply divergent mitochondrial clades (A = Central American, B = Darién, C = Chocó, D = Amazon; the precise distribution of the Darién clade is unknown and marked in dashed lines). The location of La Selva Biological Station in the Caribbean lowlands of Costa Rica, where fieldwork for this study was completed, is marked by a star. Map from [23].

We used vocal trait analysis and field playback experiments to assess species limits in the White-breasted Wood-Wren (*Henicorhina leucosticta*; hereafter WBWW) complex. WBWWs are found in humid lowland forest from eastern Mexico to central Colombia and the Chocó region of northwestern Ecuador, and also in the western and northern Amazon Basin (Fig 1). WBWWs vary subtly in plumage throughout their distribution—thirteen subspecies are currently recognized on the basis of plumage variation (Table 1)—and also show geographic variation in vocal traits including pitch and repetition of song style [19]. Two phylogenetic studies have examined geographic patterns of mtDNA in WBWWs [20,21]. Both studies identified three major clades within the WBWW complex—one in Central America, a second in the Chocó of northwest Ecuador, and a third in the Amazon basin. These three clades are deeply divergent, with estimated 7–12% mtDNA sequence divergence between clades [21]. Additional sampling in Panama revealed that birds in eastern Panama comprise a fourth distinct clade (the Darién clade) that is most closely related to the Amazon clade. The geographic distribution of this Darién clade remains uncertain, though it presumably contacts the Central American clade in central Panama [21,22] and the Chocó clade in western Colombia.

**Table 1:** Summary of subspecies and their clades based on [21]. Birds from western Colombia are presumed to be within the Chocó clade pending further evidence.

Subspecies	Range	Clade
***H*. *l*. *decolorata***	E. Mexico	Central American
***H*. *l*. *prostheleuca***	S & E Mexico	Central American
***H*. *l*. *smithei***	S Yucatán to S Guatamala	Central American
***H*. *l*. *tropaea***	Honduras and Nicaragua	Central American
***H*. *l*. *costaricensis***	N and E Costa Rica	Central American
***H*. *l*. *pittieri***	SW Costa Rica and W Panama	Central American
***H*. *l*. *alexandri***	E Panama and NW Colombia	Darién
***H*. *l*. *darienensis***	E Panama and W Colombia	Darién
***H*. *l*. *inornata***	W Colombia to NW Ecuador	Chocó
***H*. *l*. *albilateralis***	W Colombia to NW Ecuador	presumed Chocó
***H*. *l*. *eucharis***	W Colombia	presumed Chocó
***H*. *l*. *leucosticta***	E & S Venezuela, Guyana, Suriname, N Brazil	Amazon
***H*. *l*. *hauxwelli***	S. Colombia, E Ecuador, NE Peru	Amazon

We focused on the Central American, Chocó and Amazon clades for our analysis, because the geographic distributions of these clades are sufficiently well defined that audio recordings could be unequivocally identified to clade. We first analyzed differences in vocal traits using archived audio recordings, then conducted field playback experiments at La Selva Biological Station in the Caribbean lowlands of Costa Rica to measure the responses of Central American WBWWs to vocalizations from allopatric Amazonian and Chocó populations. We predicted that Central American WBWWs would show the weakest responses to Chocó playback, as Central American populations are most distantly related to the Chocó clade (mtDNA sequence divergence = 9.7–11.5%) and are slightly more closely related to Amazonian birds (mtDNA sequence divergence = 7.1–9.2%) [21]. More broadly, we predicted that differences in behavioral response to playback of allopatric populations would correlate with between-population differences in vocal traits measured from recordings. Finally, our behavioral observations and vocal analyses provide new evidence measuring premating reproductive isolation based on vocalizations between Central American WBWWs and allopatric populations. Thus, our data could generate data relevant to species limits within the WBWW complex, a taxon whose within-species systematics have heretofore only been studied using phylogenetic methods.

## Materials and Methods

### Study species

WBWWs are territorial insectivorous passerines that inhabit the low understory of Neotropical forests [24]. They are sedentary, with low dispersal rates, and individuals or mated pairs defend territories year-round [24]. Because males primarily defend territories from conspecifics using song, we assumed that responding individuals were male and our experiment used the response of these territorial birds to playback of songs as a way of measuring species recognition. At the site where we conducted our field experiments, La Selva Biological Station in Costa Rica (see below), WBWW territories average 3.8 ha in size—territory size is dependent on the amount of leaf litter available for foraging [25]. WBWWs defend their territories using song, and respond aggressively to playback of local conspecific song [26]. Previous studies demonstrate that WBWWs can respond aggressively to playback of foreign conspecific song (albeit at diminished aggression relative to local conspecific song: [27]) and even heterospecific song (e.g, to the congeneric Gray-breasted Wood-wren *Henicorhina leucophrys*) where the two species meet and appear to defend interspecific territories [26]. Thus, WBWWs, at least in some cases, respond aggressively to songs that are not from local conspecifics, and this aggression may reflect either the absence of premating reproductive isolation (to conspecific song) or ecological competition (to heterospecific song).

### Sound Analysis

**Fig 2:**
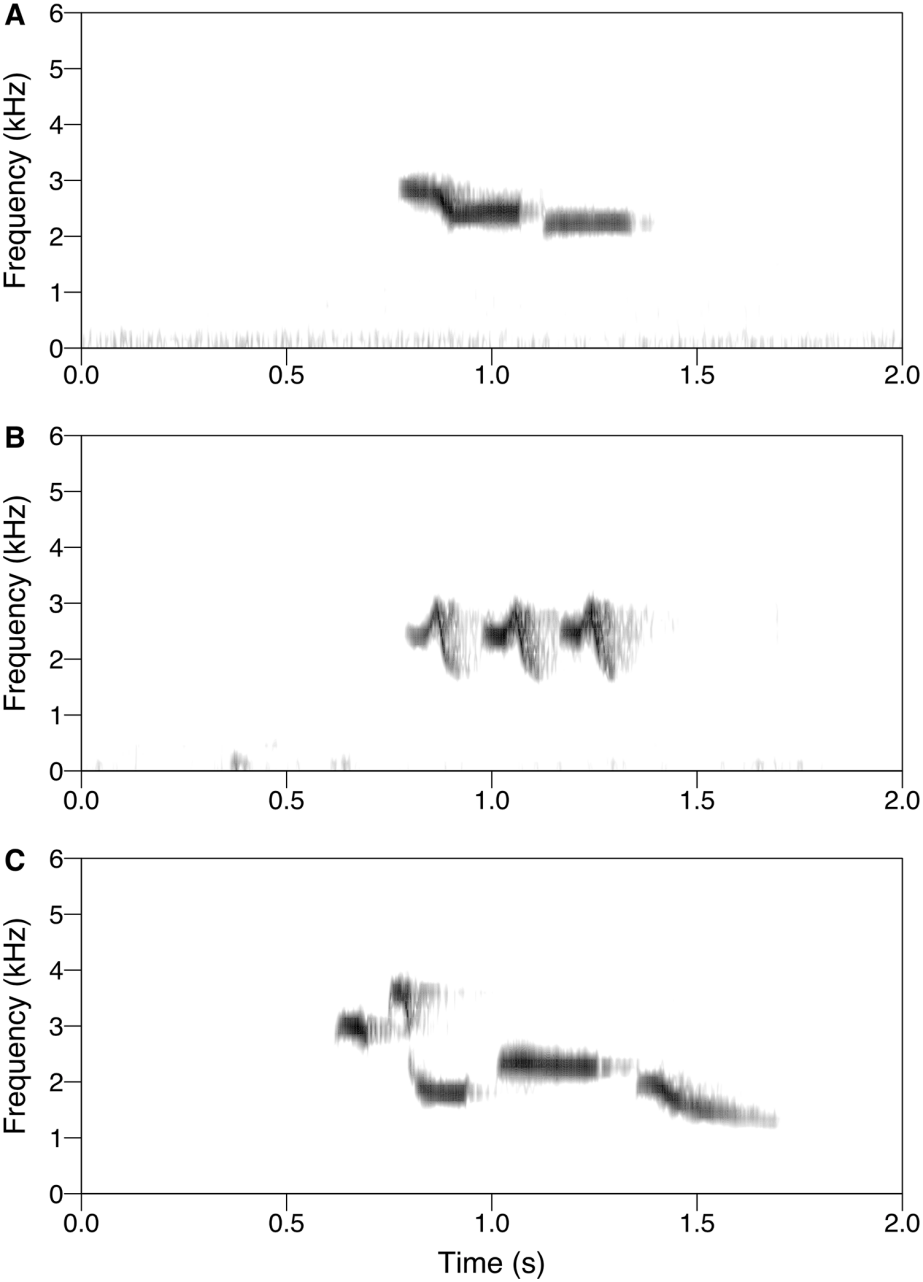
Spectrograms showing representative song phrases from White-breasted Wood-Wren populations. (A) Central American (Costa Rica, ML3922); (B) Amazon (ML46962); and (C) Chocó (XC17276).

We analyzed the songs of individuals from each of the three different clades to determine whether they had diagnosable differences in vocal traits. We analyzed a total of 49 recordings of WBWW songs (*n* = 19 from the Costa Rican population, *n* = 10 from the Chocó population in northwestern Ecuador and western Colombia, and *n* = 20 from the Amazon population) downloaded from the xeno-canto repository (http://xeno-canto.org) and the Macaulay Library of Natural Sounds (http://macaulaylibrary.org; representative spectrograms shown in Fig 2). We imported these recordings into Raven Pro 1.5 [28] and measured eight song variables (song length, total note count, mean number of notes per syllable, mean note rate, peak frequency, mean note high frequency, mean note low frequency, and total song frequency range) for a representative song from each recording, following the protocol of Mason et al [29]. All spectrogram settings in Raven were left at their defaults, except that DFT was set at 512. We defined a note as a discrete sound preceded and followed by silence in the spectrogram. We visualized population differences in vocal traits using the first two axes from a principal components analysis (PCA) that quantified variation across the nine vocal characters (Table 2), and used a discriminant function analysis (DFA) to quantify our ability to predict the population from which a given recording was sampled given its measured vocal traits. We used half the data to train the DFA and the other half of the data to quantify classification rates of the DFA. A list of the recordings used in our playback and sound analyses can be found in S1 Table.

**Table 2:** Summary of vocal traits for three populations of WBWWs. Vocal trait values are presented as mean +/- standard deviation.

Population	Sample Size	Peak Song Freq. (Hz)	Note High Freq. (Hz)	Note Low Freq. (Hz)	Song Freq. Range (Hz)	Mean Song Length (s)	Mean Note Length (s)	Note Rate (notes/s)	Note Count
Amazon	20	2978 +/- 685	5688 +/- 1871	1782 +/- 361	3906 +/- 1931	1.05 +/- 0.28	0.12 +/- 0.04	6.89 +/- 1.73	7.25 +/- 2.81
Chocó	10	3377 +/- 763	4580 +/- 779	1519 +/- 265	3061 +/- 822	1.02 +/- 0.15	0.18 +/- 0.06	4.88 +/- 1.19	4.90 +/- 0.88
Costa Rica	19	2656 +/- 567	3183 +/- 622	1835 +/- 298	1347 +/- 456	0.79 +/- 0.16	0.22 +/- 0.04	4.09 +/- 0.67	3.21 +/- 0.63

### Field experiments

We conducted field experiments on a single population of WBWWs at La Selva Biological Station, Heredia province, in the Caribbean lowlands of northeastern Costa Rica, where WBWWs are abundant [30]. WBWWs are not an endangered or protected species, and we did not physically manipulate any individuals. Thus, no permits were required for the described study, which complied with the Guidelines to the Use of Wild Birds in Research and was carried out with the permission of the Organization for Tropical Studies (OTS), the owner of La Selva Biological Station. We performed two separate playback experiments on WBWWs. Each experiment contained two treatments; a sympatric treatment of local Costa Rican song playback (*H*. *l*. *costaricensis*) and an allopatric treatment of song playback from a different population. One experiment (hereafter the “Amazon experiment”) used playback of vocalizations from WBWWs from Amazonia as the allopatric treatment (*H*. *l*. *hauxwelli*) whereas the other (hereafter the “Chocó experiment”) used playback of Chocó clade vocalizations (*H*. *l*. *inornata*) as the allopatric treatment. Trials for these two experiments were carried out on different WBWW territories at La Selva and analyzed independently. The recordings of natural vocalizations of WBWWs we used for experiments are archived at xeno-canto.org or the Macaulay Library of Natural Sounds. We confirmed the provenance of the sounds we analyzed and used for fieldwork by our own expert analysis. The function of WBWW songs is little studied, but it is likely that WBWW songs function both in territorial defense and mate choice [24] (see Discussion for further consideration of this issue). We minimized the potential problem of pseudoreplication by using multiple natural vocalizations of primary songs from each population (*n* = 4 for sympatric treatments and *n* = 5 for allopatric treatments; see S1 Table).

We performed the majority of experiments in the morning hours (6:00 am – 12:00 pm), but included some trials in the afternoon and evening (12:00 pm – 6:00 pm). The mean and median trial times were both approximately 10:00 am. We located WBWW territories while walking forest trails at La Selva. Birds were typically initially located by direct observation—a male bird was either heard singing or visually observed in the understory. Occasionally we broadcast brief snippets of local WBWW song (< 10 s at a time) to discover territories of birds that had not been previously detected. After locating a territory, we began a playback trial, though we did not begin playback trials if the bird we were targeting was actively singing—in such instances we waited until two minutes after the individual stopped singing to initiate a playback experiment.

We used a Pignose amplifier (hereafter “speaker”) to broadcast song. The speaker was attached to a 12 m long cable, allowing us to stand around 10 m away from the speaker during experiments, minimizing the possibility that responding individuals would be influenced by our presence. Each experimental trial consisted of two minutes of playback of the first treatment followed by a five-minute observation period, then two minutes of the second playback treatment, and a five-minute observation period. We alternated the order of treatments between experimental trials (allopatric treatment first vs. sympatric treatment first), which allowed us to test for playback sequence effects. Both treatments were performed successively on each territory, and most treatments were in direct succession, resulting in an experimental trial that lasted fourteen minutes. However, if a territorial bird was still vocalizing in response to the first playback treatment at the end of the five-minute observation period, we waited an extended period—until two minutes after it stopped vocalizing—prior to beginning the second treatment. During the observation periods, multiple observers recorded the following behavioral responses: latency to approach (s), closest approach to the speaker (m), latency to vocalize (s), and number of songs. Importantly, the sympatric treatment in each experiment functioned as a positive control—we expected birds to respond strongly to sympatric playback. We did not include a negative control in our experiments, as our primary goal was to compare responses of WBWWs to playback of songs from two different allopatric populations (Chocó vs. Amazon).

Territorial males often engage in countersinging behavior, and neighboring males sing to each other from within their own territories. However, when a rival invades the territory of a male, the territory owner will often approach the invader to drive him away. Therefore, while song responses alone do not necessarily indicate that a speaker is on a territory, if a male approaches the speaker during sympatric playback, we inferred that the male perceived the playback stimulus to represent a conspecific. Thus, we assume that an approach response is more aggressive than a song response. Supporting this assumption, birds approached the speaker in response to sympatric playback in all successful experiments.

We analyzed approaches to the speaker in two ways. First, we classified individuals based on whether or not they approached to 15 m away (or closer) from the speaker in response to playback treatments (yes/no). Second, we measured how close birds approached the speaker in response to playback treatments. When a bird did not approach the speaker in response to an allopatric playback, we coded its approach distance as 15 m, the approximate distance from the speaker within which we could confidently observe approaching territorial individuals that remained silent (given understory vegetation). When a bird did not sing in response to playback, we coded latency to vocalize as 420 s (equal to seven minutes, or the length of an entire trial).

We performed all statistical tests in R version 3.1.2 [31]. The two experiments (Chocó and Amazon) were analyzed separately. Because data were determined to be non-normal (by visual assessment), we compared all continuous variables using paired Wilcoxon rank-sum tests, comparing the response of each individual to the allopatric recording to the response of the same individual to the sympatric recording. We compared the probability that birds approached the speaker (yes/no) between treatment groups using a test of equal proportions (hereafter referred to as “p-test”). Lastly, we assessed whether the order of sympatric and allopatric playback, or the time of day of each trial, had any significant effect on trial outcome.

## Results

### Vocal trait analysis

**Fig 3:**
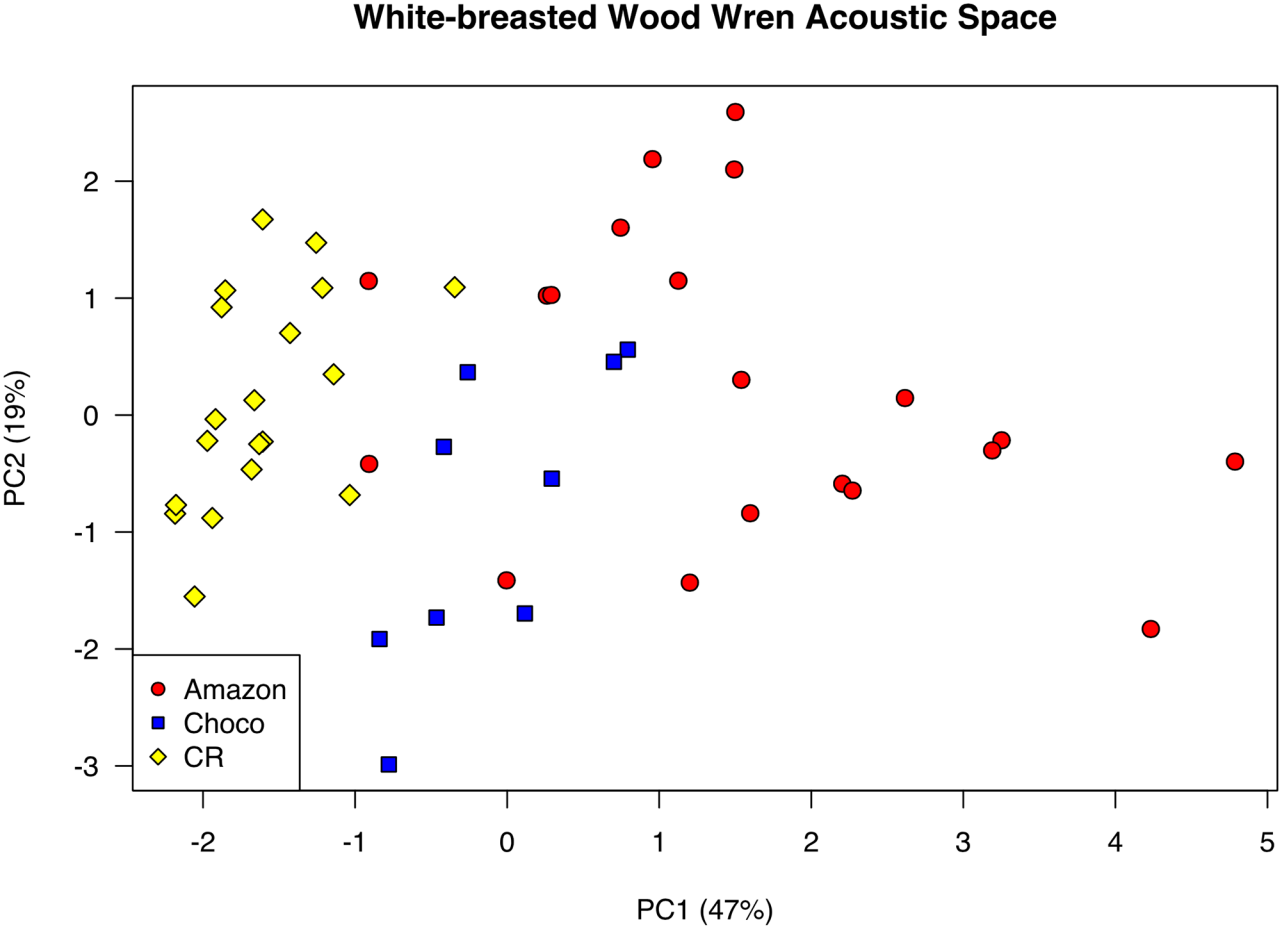
PCA plot of acoustic space. Larger values on PC1 represent songs that are longer, that have more notes (that are shorter) and that cover a wider range of frequencies. Larger values on PC2 represent songs with faster note rates and smaller frequency ranges. Populations, shown in yellow (Costa Rica), red (Amazon) and blue (Chocó), are somewhat differentiated in acoustic space.

Vocal traits varied between WBWW populations. For example, on average, songs from Costa Rican birds were shorter and covered a narrower frequency range than those from the Chocó and Amazon, while Amazonian songs had a faster note rate and a greater number of notes (Table 2). However, there was considerable within-region variation in vocal traits (note the large standard deviations for most traits, Table 2). The first axis of the PCA on vocal traits explained 47% of variation in the data, with song length, note rate, note count and two frequency variables (high frequency and song frequency range) all loading positively on PC1 (loadings = 0.32–0.46; see Table 3), while mean note length loaded negatively (= -0.39). PC2 explained an additional 19% of variation, with note rate and note low frequency loading positively (loadings = 0.45 and 0.42, respectively), and mean note length, high frequency, song length, and song frequency range all loading negatively (loadings = -0.27 to -0.49). Populations showed a clear clustering trend along PC1 in a plot of the first two PC axes (a graphical depiction of “acoustic space”, Fig 3); songs from Costa Rica individuals were tightly clustered with negative PC1 values (-1.84 +/- 0.57, PC1 mean +/- standard deviation), while songs from Chocó (-0.090 +/- 0.59) and especially Amazonian (1.70 +/- 1.53) individuals were more differentiated (Fig 3). We further quantified this pattern using a discriminant function analysis (model runs = 1000), which on average correctly classified 93.6% of Costa Rican recordings (sd = 4.6%), 68.2% of Choco recordings (sd = 12.5%), and 89.6% of Amazonian recordings (sd = 6.7%). Thus, while populations were differentiated in acoustic space (particularly between Costa Rican and Amazonian populations), there was also sufficient overlap such that vocalizations could not be diagnosably assigned to population.

**Table 3:** Loading scores for the first two principal components (PC1 and PC2, which explained 47% and 19% of variation, respectively) from the vocal trait analysis.

Vocal Trait	PC1	PC2
Peak song frequency (Hz)	0.164	-0.132
Note high frequency (Hz)	0.397	-0.276
Note low frequency (Hz)	-0.085	0.423
Song frequency range (Hz)	0.406	-0.352
Mean song length (s)	0.322	-0.379
Mean note length (s)	-0.392	-0.491
Note rate (notes/s)	0.413	0.453
Note count	0.465	0.113

### Field experiments

**Fig 4:**
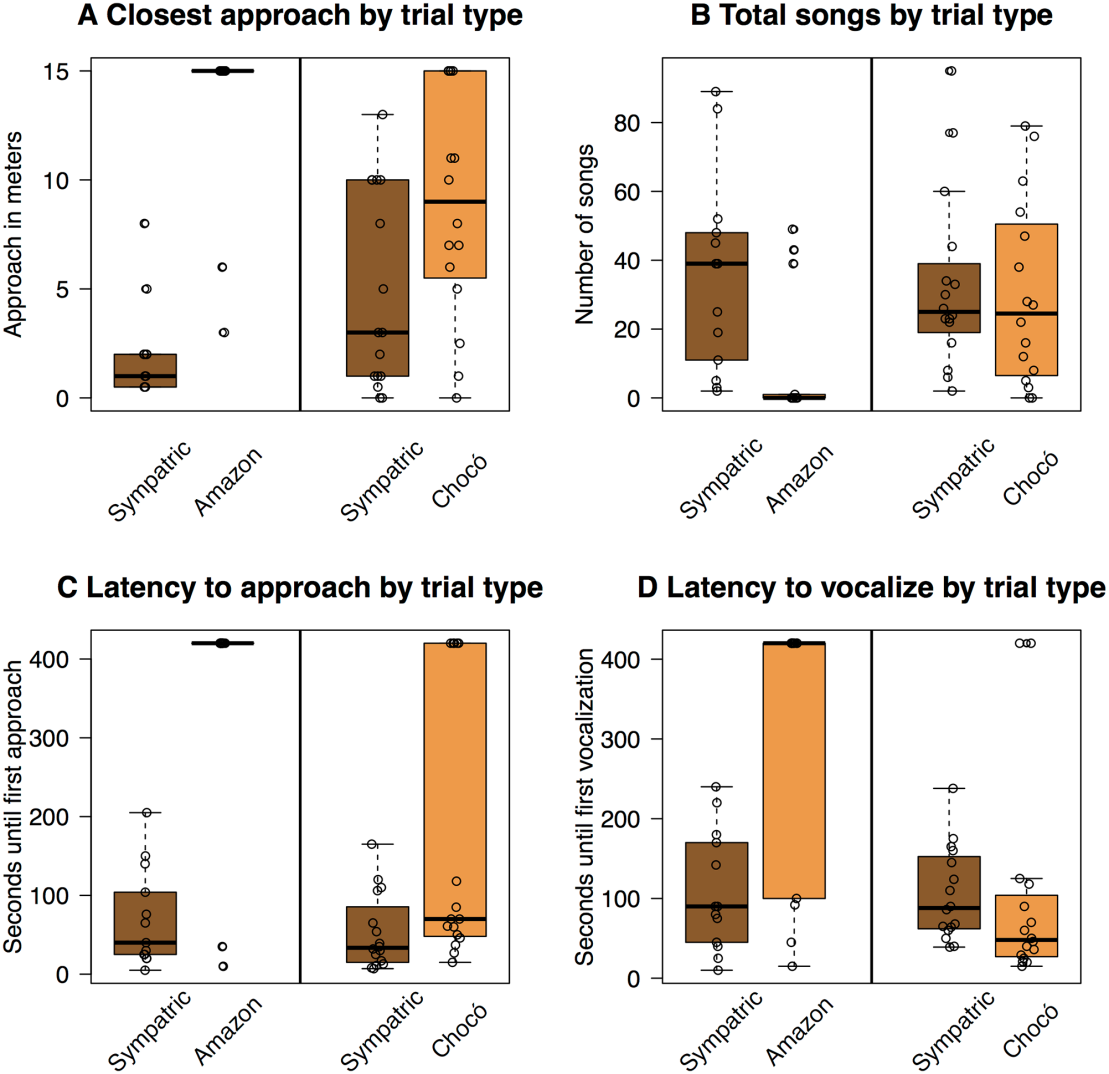
Behavioral response to playback experiments. Responses to the Amazon experiment are on the left and responses to the Chocó experiment are on the right. Latency to approach (A), closest approach to speaker (B), total number of songs (C) and latency to vocalize (D). Boxplots illustrate median (horizontal black bar), first and third quartiles (boxes), and minimum and maximum values (points and dotted lines). Raw data are plotted as points in front of the boxplots, with points offset slightly to better display values. Sympatric treatments elicited aggressive responses (fast approaches, close approaches, many songs, low latency to vocalize) in both experiments. Allopatric treatments elicited asymmetric responses: individuals typically responded aggressively to playback of Chocó songs but not to playback of Amazonian songs.

**Fig 5:**
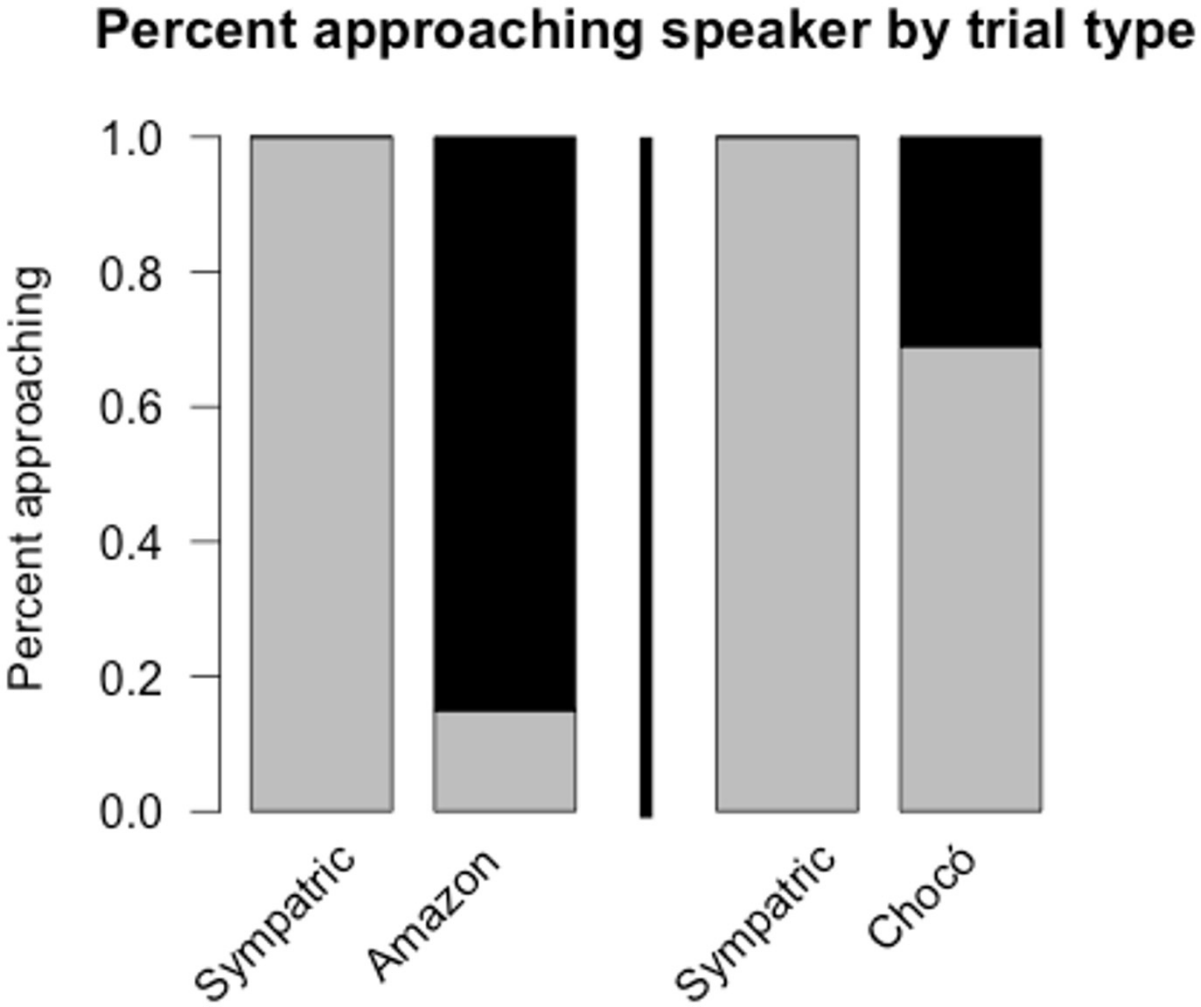
Percent of individuals approaching the speaker in each experiment. Responses to the Amazon experiment are on the left and responses to the Chocó experiment are on the right. Note that sympatric trials serve as positive controls (wrens approached the speaker in all sympatric trials).

WBWWs responded strongly to sympatric treatments in both experiments, typically approaching the speaker quietly in response to sympatric playback before vocalizing loudly for several minutes. Within both experiments, the response to the allopatric treatment was weaker than to the sympatric treatment. For example, WBWWs in both experiments approached the speaker sooner during sympatric playback than allopatric playback (Fig 4A) and they also tended to come closer to the speaker during sympatric playback (Fig 4B). However, birds often did not approach at all in response to allopatric playback, and in this regard there was a strong difference between the two experiments: In the Chocó experiment, 69% (11 out of 16) of individuals approached the speaker in response to Chocó playback, whereas in the Amazon experiment, only 15% (2 out of 13) of individuals approached the speaker in response to Amazon playback (Fig 5). Similarly, WBWWs gave much stronger vocal responses to Chocó playback than to Amazon playback. The number of songs in response to Chocó playback (30 ± 27 songs; mean ± sd) did not differ significantly from the response to sympatric playback (33 ± 25 songs, p = 0.31); but in the Amazon experiment, birds gave significantly more songs in response to sympatric playback (35 ± 29 songs) than they did to Amazon playback (10 ± 19 songs, p = 0.04) (Fig 4C). In addition, latency to vocalize to playback of Chocó vocalizations (99 ± 130 s) was not significantly different than that following sympatric playback (105 ± 58 s, p = 0.2; Fig 4D), while latency to vocalize to allopatric Amazon playback (310 ± 173 s) was significantly longer than that for sympatric playback (108 ± 75 s, p = 0.0097).

We also tested whether playback sequence and time of day influenced our results. Each trial consists of two playback treatments (sympatric and allopatric), with the sequence of treatments alternated between trials. We compared responses to first-round playback and second-round playback (regardless of playback type) and found no effect of playback sequence on any of our four responses (latency to approach, approach distance, latency to vocalize, or number of songs; all p> 0.1). We also used linear models to test for associations between the time of a trial and our four response variables, and again found no significant results (all p > 0.1). Thus, neither playback sequence nor time of day influenced our results.

## Discussion

Our field experiment revealed asymmetric responses of Costa Rican WBWWs to playback from two different allopatric populations—WBWWs from Costa Rica, from the Central American clade, responded strongly to song playback from the Chocó clade but only weakly to song playback from the Amazon clade. This difference in behavioral response to field experiments is consistent with our vocal trait analysis, which showed songs from the Central American clade to be closest in acoustic space to the Chocó clade and furthest in acoustic space from the Amazon clade. While the results of our playback experiments are consistent with the vocal trait analysis, we note that, in the vocal trait analysis, the three populations were not diverged enough to be diagnosably different in acoustic space (as defined by a PCA using commonly measured song attributes), suggesting that using these methods in combination allows for a more robust test of vocalization-based reproductive isolation than either by itself.

Our data are consistent with previous suggestions that the WBWW complex consists of multiple biological species [20,21]. The Chocó clade is the most genetically divergent clade in the WBWW complex, with the Central American population more closely related to (though still deeply divergent from) the Amazon clade than the Chocó clade [20,21]. The strong response of Costa Rican birds to Chocó playback suggests that the Costa Rica subspecies likely recognizes Chocó WBWWs as vocally conspecific, while the lack of response to Amazon playback suggests that Costa Rican WBWWs generally do not recognize Amazonian WBWWs as vocally conspecific. This result implies that, despite the extensive divergence in mtDNA between clades, premating reproductive isolation based on song has not evolved between all allopatric populations of WBWWs (i.e., between Central American and Chocó clades). However, these results are consistent with premating reproductive isolation based on song having evolved between the Central American and Amazonian clades, suggesting that the Amazonian population of WBWWs represent a distinct biological species from birds in the Central American and Chocó clades. Reciprocal playback experiments measuring how Chocó and Amazonian populations respond to Central American songs are needed to test these ideas.

Species limits in the remainder of the WBWW complex remain unclear. The genetic distance between Central American and Chocó clades (9.7–11.5% divergence in mtDNA sequences) [21] is sufficiently large that postmating reproductive isolation may have evolved between these two clades [7], such that the Chocó clade represents a distinct biological species. We found a relative lack of premating reproductive isolation between the Central American and Chocó clades based on song playback, but we did document a reduced response to playback of Chocó songs relative to sympatric songs, which potentially indicates some degree of premating reproductive isolation. Other traits relevant to premating reproductive isolation that we did not measure (e.g. plumage, which differs slightly between different subspecies) may also have evolved. Further study of the Darién clade (and its presumed contact zones with the Central American clade in central Panama [21,22] and the Chocó clade in western Colombia) using genomic tools, audio recordings and playback experiments could determine whether or not the mitochondrial, vocal and plumage divergence between clades constitute a strong barrier to gene flow at these contact zones. In addition, the genetic relationships of WBWWs in central Colombia are unknown and may shed further light on the WBWW complex’s evolutionary history.

We found a stronger behavioral response by Costa Rican WBWWs to song playback of the more distantly related Chocó clade (see [15]). Understanding why song evolution is uncorrelated with genetic distance in this case is beyond the scope of this study. This lack of correlation could arise for several nonexclusive reasons, including: 1) song differences between allopatric WBWW populations evolve in a drift-like fashion such that genetic distance is only loosely correlated with song divergence; 2) the acoustic environment of the Amazon is different from that of the Chocó and Costa Rica and has selected for different song characteristics; and 3) the modern biogeographic connection between the Chocó and Costa Rica (via the Isthmus of Panama) has permitted cultural transmission of song between populations west of the Andes and has thus reduced or prevented song divergence between these two populations and the Darién clade relative to the Amazonian clade.

It is important to note several caveats to our conclusions. First, we did not test reciprocal responses of Amazon and Chocó birds to playback of Central American birds. Responses to playback are not always symmetrical between populations [32]—we interpret the lack of Central American WBWW response to Amazonian WBWW playback as evidence of premating reproductive isolation between these two populations. Further studies should measure how Amazonian WBWWs respond to vocalizations from Central American WBWWs; given the marked differences in vocal traits, we predict that Amazonian WBWWs are unlikely to respond strongly to Central American WBWW vocalizations. Second, we tested a territorial response rather than a mate choice response. In general, research suggests that females are more discriminating than males when it comes to songs, implying that if males discriminate between songs, as we demonstrate, it is likely that females do as well [33–37]. All vocal trait analyses and playback experiments aimed at determining species limits make the assumption that territorial songs and mate choice are correlated, and though this is generally accepted as a valid assumption, it may not be true in all cases, especially in species with multiple song types where certain types may be used for territoriality and others for mate attraction. WBWWs are not known to have multiple song types for different purposes, but this has never been experimentally assessed. Even species with only a single song type may show a lack of tight correlation between territoriality and mate choice [38]: for example, male responses to playback (including responses to heterospecific song) can also be related to competition (e.g. [39,40]) and in some cases, defense of interspecific territories may drive convergence of song types between heterospecifics [39]. In an ideal situation, mate choice experiments measuring the response of breeding condition females to competing (sympatric vs. allopatric) song playbacks would be the best way of answering these questions. We did not conduct such experiments because capturing female individuals was beyond the scope of this study. In addition, such experiments would require sexing individuals based on phenotype (likely difficult for WBWWs) and identifying females in breeding condition (difficult for tropical species with extended or idiosyncratic breeding seasons [41,42]. Finally, we did not include the fourth clade of WBWWs—the Darién clade found in eastern Panama (and presumably adjacent Colombia) that is most closely related to the Amazon clade—as the geographic limits of this clade remain unclear.

## Conclusions

Our playback experiments are a case study demonstrating how behavioral research can provide data relevant to avian systematics. We found that, in the WBWW complex, phylogenetics, vocal traits and behavioral experiments each paint a slightly different picture of the complex history and current levels of reproductive isolation of WBWW populations throughout their range. Phylogenetic studies depict how gene flow between populations occurred in the past, and have identified four discrete mitochondrial clades within the WBWW complex [20,21]. In contrast, vocal trait analysis and behavioral experiments are methods to measure modern levels of premating reproductive isolation in vocalizations between allopatric populations. Our vocal trait analysis demonstrated divergence between populations (particularly between Central American and Amazonian populations), though populations were not unequivocally diagnosable. This pattern of vocal divergence was echoed in our field behavioral experiments: Central American WBWWs responded aggressively to songs from Chocó populations, but did not respond to songs from Amazonian populations, suggesting that Central American populations have evolved premating reproductive isolation to Amazonian populations.

Similar playback studies have shed light on systematics in various passerines [43–46] but are outnumbered by studies that only analyze vocal traits. We found clear differences in response to song playback of allopatric populations, supporting the utility of playback experiments to measure premating reproductive isolation based on song in this species complex and potentially other oscine passerines that learn songs. Thus, for territorial species that use song to attract mates, whether suboscines or oscines, playback experiments are an excellent method to test hypotheses about species limits between allopatric populations. Our study species is an especially appropriate candidate for this type of analysis—WBWWs are highly vocal, abundant, understory-dwelling, and territorial. Even so, we suggest that playback experiments can be more broadly applied to assess whether premating barriers to gene flow have developed between allopatric populations. The wide availability of song recordings from online libraries, in addition to affordable equipment for conducting song playbacks, make playback experiments easier to perform than ever before. Establishing species limits between allopatric populations remains one of the thorniest issues in modern avian systematics, and assessing premating reproductive isolation is a key issue [47,48]. We expect playback experiments will be an important tool used to delineate species limits in these cases—when it is unclear if allopatric populations have evolved song-based premating reproductive isolation, there is no better way to answer these questions than to ask the birds themselves.

## Supporting Information

S1 Script R code for statistical analysis. (R)

S1 Spreadsheet Raw data from field experiments. (CSV)

S1 Table Recordings used in playback experiments and vocal trait analysis. (PDF)
