# Supplementary material for: Asymmetric Response of Costa Rican White-Breasted Wood-Wrens (Henicorhina leucosticta) to Vocalizations from Allopatric Populations
Source: PLoS One. 2015 Dec 15;10(12):e0144949. doi: 10.1371/journal.pone.0144949 (PMC4679390; doi:10.1371/journal.pone.0144949)
Supplement: S1 Table — (PDF) [file pone.0144949.s003.pdf]

Track Number	Source	Subspecies	Clade	Locality	Used In
**XC72717**	xeno-canto	H. l. hauxwelli	Amazon	E Ecuador	Playback and PCA/DFA
**XC98005**	xeno-canto	H. l. hauxwelli	Amazon	E Ecuador	Playback and PCA/DFA
**XC216025**	xeno-canto	H. l. hauxwelli (presumed)	Amazon	N Peru	Playback and PCA/DFA
**ML17514**	Macaulay Library	H. l. hauxwelli (presumed)	Amazon	N Peru	Playback only
**ML28559**	Macaulay Library	H. l. hauxwelli (presumed)	Amazon	E Ecuador	Playback only
**XC180481**	xeno-canto	H. l. costaricensis (presumed)	Central American	Costa Rica	Playback only
**XC224085**	xeno-canto	H. l. costaricensis (presumed)	Central American	Costa Rica	Playback only
**ML76623**	Macaulay Library	H. l. costaricensis (presumed)	Central American	Costa Rica	Playback and PCA/DFA
**ML184781**	Macaulay Library	H. l. costaricensis (presumed)	Central American	Costa Rica	Playback and PCA/DFA
**XC224079**	xeno-canto	H. l. inornata (presumed)	Chocó	W Ecuador (Esmeraldas)	Playback and PCA/DFA
**XC85527**	xeno-canto	H. l. inornata	Chocó	W Ecuador (Esmeraldas)	Playback and PCA/DFA
**XC19519**	xeno-canto	H. l. inornata	Chocó	W Ecuador (Esmeraldas)	Playback only
**XC17276**	xeno-canto	H. l. inornata	Chocó	W Ecuador (Esmeraldas)	Playback and PCA/DFA
**XC76403**	xeno-canto	H. l. inornata	Chocó	W Ecuador (Esmeraldas)	Playback only
**ML46962**	Macaulay Library	H. l. leucosticta (presumed)	Amazon	Brazil (Amazonas)	PCA/DFA only
**ML53746**	Macaulay Library	H. l. hauxwelli (presumed)	Amazon	E Ecuador	PCA/DFA only
**ML79712**	Macaulay Library	H. l. hauxwelli (presumed)	Amazon	S Ecuador	PCA/DFA only
**ML79715**	Macaulay Library	H. l. hauxwelli (presumed)	Amazon	S Ecuador	PCA/DFA only
**ML90435**	Macaulay Library	H. l. hauxwelli (presumed)	Amazon	E Ecuador	PCA/DFA only
**XC12867**	xeno-canto	H. l. hauxwelli	Amazon	E Ecuador	PCA/DFA only
**XC17846**	xeno-canto	H. l. hauxwelli	Amazon	E Ecuador	PCA/DFA only

Track Number	Source	Subspecies	Clade	Locality	Used In
**XC189213**	xeno-canto	H. l. hauxwelli	Amazon	S Ecuador	PCA/DFA only
**XC190204**	xeno-canto	H. l. hauxwelli	Amazon	S Ecuador	PCA/DFA only
**XC207949**	xeno-canto	H. l. hauxwelli	Amazon	S Ecuador	PCA/DFA only
**XC209073**	xeno-canto	H. l. hauxwelli (presumed)	Amazon	S Ecuador	PCA/DFA only
**XC4230**	xeno-canto	H. l. hauxwelli (presumed)	Amazon	E Ecuador	PCA/DFA only
**XC4880**	xeno-canto	H. l. hauxwelli (presumed)	Amazon	E Ecuador	PCA/DFA only
**XC72715**	xeno-canto	H. l. hauxwelli	Amazon	E Ecuador	PCA/DFA only
**XC8080**	xeno-canto	H. l. hauxwelli	Amazon	E Ecuador	PCA/DFA only
**XC9328**	xeno-canto	H. l. hauxwelli (presumed)	Amazon	E Ecuador	PCA/DFA only
**XC98006**	xeno-canto	H. l. hauxwelli	Amazon	E Ecuador	PCA/DFA only
**ML48912**	Macaulay Library	H. l. inornata (presumed)	Chocó	W Ecuador (Esmeraldas)	PCA/DFA only
**ML48944**	Macaulay Library	H. l. inornata (presumed)	Chocó	W Ecuador (Esmeraldas)	PCA/DFA only
**ML63408**	Macaulay Library	H. l. inornata (presumed)	Chocó	W Ecuador (Esmeraldas)	PCA/DFA only
**XC11959**	xeno-canto	H. l. inornata	Chocó	W Ecuador (Esmeraldas)	PCA/DFA only
**XC224080**	xeno-canto	H. l. inornata (presumed)	Chocó	W Ecuador (Esmeraldas)	PCA/DFA only
**XC85526**	xeno-canto	H. l. inornata	Chocó	W Ecuador (Esmeraldas)	PCA/DFA only
**ML184032**	Macaulay Library	H. l. costaricensis (presumed)	Central American	Costa Rica	PCA/DFA only
**ML184033**	Macaulay Library	H. l. costaricensis (presumed)	Central American	Costa Rica	PCA/DFA only
**ML184036**	Macaulay Library	H. l. costaricensis (presumed)	Central American	Costa Rica	PCA/DFA only
**ML184053**	Macaulay Library	H. l. costaricensis (presumed)	Central American	Costa Rica	PCA/DFA only
**ML184104**	Macaulay Library	H. l. costaricensis (presumed)	Central American	Costa Rica	PCA/DFA only

Track Number	Source	Subspecies	Clade	Locality	Used In
**ML184106**	Macaulay Library	H. l. costaricensis (presumed)	Central American	Costa Rica	PCA/DFA only
**ML184200**	Macaulay Library	H. l. costaricensis (presumed)	Central American	Costa Rica	PCA/DFA only
**ML184708**	Macaulay Library	H. l. costaricensis (presumed)	Central American	Costa Rica	PCA/DFA only
**ML184712**	Macaulay Library	H. l. costaricensis (presumed)	Central American	Costa Rica	PCA/DFA only
**ML184767**	Macaulay Library	H. l. costaricensis (presumed)	Central American	Costa Rica	PCA/DFA only
**ML39222**	Macaulay Library	H. l. costaricensis (presumed)	Central American	Costa Rica	PCA/DFA only
**ML72612**	Macaulay Library	H. l. costaricensis (presumed)	Central American	Costa Rica	PCA/DFA only
**ML74099**	Macaulay Library	H. l. costaricensis (presumed)	Central American	Costa Rica	PCA/DFA only
**ML74122**	Macaulay Library	H. l. costaricensis (presumed)	Central American	Costa Rica	PCA/DFA only
**ML74148**	Macaulay Library	H. l. costaricensis (presumed)	Central American	Costa Rica	PCA/DFA only
**ML76621**	Macaulay Library	H. l. costaricensis (presumed)	Central American	Costa Rica	PCA/DFA only
**ML76622**	Macaulay Library	H. l. costaricensis (presumed)	Central American	Costa Rica	PCA/DFA only
